# Procedural virtual reality simulation training for robotic surgery: a randomised controlled trial

**DOI:** 10.1007/s00464-020-08197-w

**Published:** 2021-01-04

**Authors:** Nicholas Raison, Patrick Harrison, Takashige Abe, Abdullatif Aydin, Kamran Ahmed, Prokar Dasgupta

**Affiliations:** 1 Division of Transplantation Immunology and Mucosal Biology, Faculty of Life Sciences and Medicine, MRC Centre for Transplantation, King’s College London, Guy’s Hospital, 5th Floor Tower Wing, London, SE1 9RT UK; 2 Department of Urology, Hokkaido University Graduate School of Medicine, Sapporo, Japan

**Keywords:** Robotic surgery, Surgical education, Simulation, Virtual reality

## Abstract

**Background:**

Virtual reality (VR) training is widely used for surgical training, supported by comprehensive, high-quality validation. Technological advances have enabled the development of procedural-based VR training. This study assesses the effectiveness of procedural VR compared to basic skills VR in minimally invasive surgery.

**Methods:**

26 novice participants were randomised to either procedural VR (*n* = 13) or basic VR simulation (*n* = 13). Both cohorts completed a structured training programme. Simulator metric data were used to plot learning curves. All participants then performed parts of a robotic radical prostatectomy (RARP) on a fresh frozen cadaver. Performances were compared against a cohort of 9 control participants without any training experience. Performances were video recorded and assessed blindly using GEARS post hoc.

**Results:**

Learning curve analysis demonstrated improvements in technical skill for both training modalities although procedural training was associated with greater training effects.

Any VR training resulted in significantly higher GEARS scores than no training (GEARS score 11.3 ± 0.58 vs. 8.8 ± 2.9, *p* = 0.002). Procedural VR training was found to be more effective than both basic VR training and no training (GEARS 11.9 ± 2.9 vs. 10.7 ± 2.8 vs. 8.8 ± 1.4, respectively, *p* = 0.03).

**Conclusions:**

This trial has shown that a structured programme of procedural VR simulation is effective for robotic training with technical skills successfully transferred to a clinical task in cadavers. Further work to evaluate the role of procedural-based VR for more advanced surgical skills training is required.

**Electronic supplementary material:**

The online version of this article (10.1007/s00464-020-08197-w) contains supplementary material, which is available to authorized users.

Training simulators trace their origins to the mechanical Link trainer developed in 1929 for pilots to practice flying by instruments. It was not until 1993 that Richard Satava et al. [1] developed the first medical VR simulator. Since then, advances in computer hardware and software have continued to drive the development of ever more realistic and complex VR surgical simulators. Currently, the majority of VR trainers, especially for laparoscopic and robotic surgery, offer basic surgical skills training for core motor skills. In robotic surgery they include endowrist manipulation, clutching, three-dimensional vision, dexterity, tissue handling, instrument control and camera control. VR modules mostly use abstract exercises such as placing hoops on pegs or manipulating objects for specific skills.

Whilst VR simulation is acknowledge as being effective in teaching basic surgical skills [2], bridging the gap between such isolated skills training and undertaking full surgical procedures in the operating room (OR) requires further extensive training.

Advances in software and hardware have led to the development of increasingly realistic VR environments. Procedural-based simulation aims to extend training beyond abstract tasks and recreate complete or part of a surgical procedure. This allows training of both basic and advanced skills such as managing bleeding. Even for basic surgical VR, modelling elements such as shadows, the effects of collision, and topological changes due to tearing, grasping or cutting is challenging. With procedural VR, the complexity is greatly increased with the need to accommodate surgical factors such as the effects of instruments or sutures on tissues, physiological responses such as bleeding and accurate anatomical modelling [3]. The simulator should also be able to provide useful and objective assessments of performance for training and assessment. Procedural VR training has been shown to be effective in training laparoscopic cholecystectomy and salpingectomy [4, 5]. A number of robotic VR simulators offer procedural or part procedural training, however, to date assessment of the effectiveness of this training has been limited. The RobotiX Mentor (3D Systems, Airport City, Israel) offers seven different procedural training modules covering gynaecological, thoracic, urological and general surgery.

This study aims to compare the effectiveness of structured procedural VR training against basic VR training and no training for robotic surgery.

## Methods

A multi-institutional, randomised controlled trial was conducted in the Vattikuti Institute for Robotic Surgery, King’s College London. Data were collected between the March and November 2016.

**Fig. 1:**
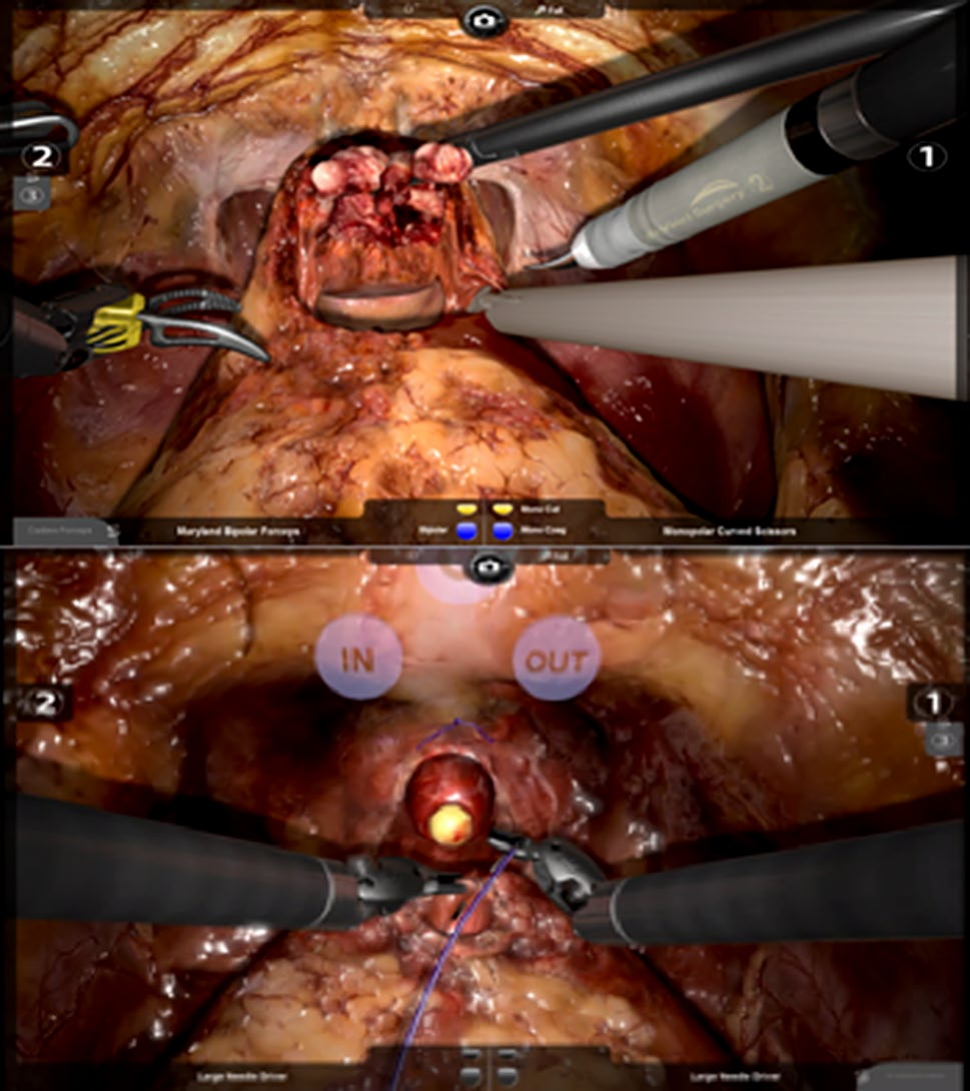
Example of BND and UVA RobotiX mentor training modules

The RobotiX Mentor robot-assisted radical prostatectomy (RARP) module was selected for this study. Alongside video-based didactic training, hands-on VR training can be undertaken either with or without step-by-step procedural guidance. Guided training modules were used throughout the study. The module consists of four training tasks; bladder neck dissection (BND), neurovascular bundle dissection, apical dissection and urethrovesical anastomosis (UVA) (Fig. 1). Given that the neurovascular bundle and apical dissection tasks require specialist anatomical knowledge for successful completion, it was decided by the authors to exclude these modules. The primary outcome measure was the operative technical performance, assessed on fresh frozen cadaveric models within a simulated operating room. The secondary outcome measure was the training effect evaluated through learning curve analysis.

### Subjects

Novice participants (without any experience in robotic surgery) were recruited by open invitation from London medical schools (King’s College London; Bart’s and The London School of Medicine and Dentistry; Imperial College School of Medicine; University College London Medical School).

### Training programme

**Fig. 2:**
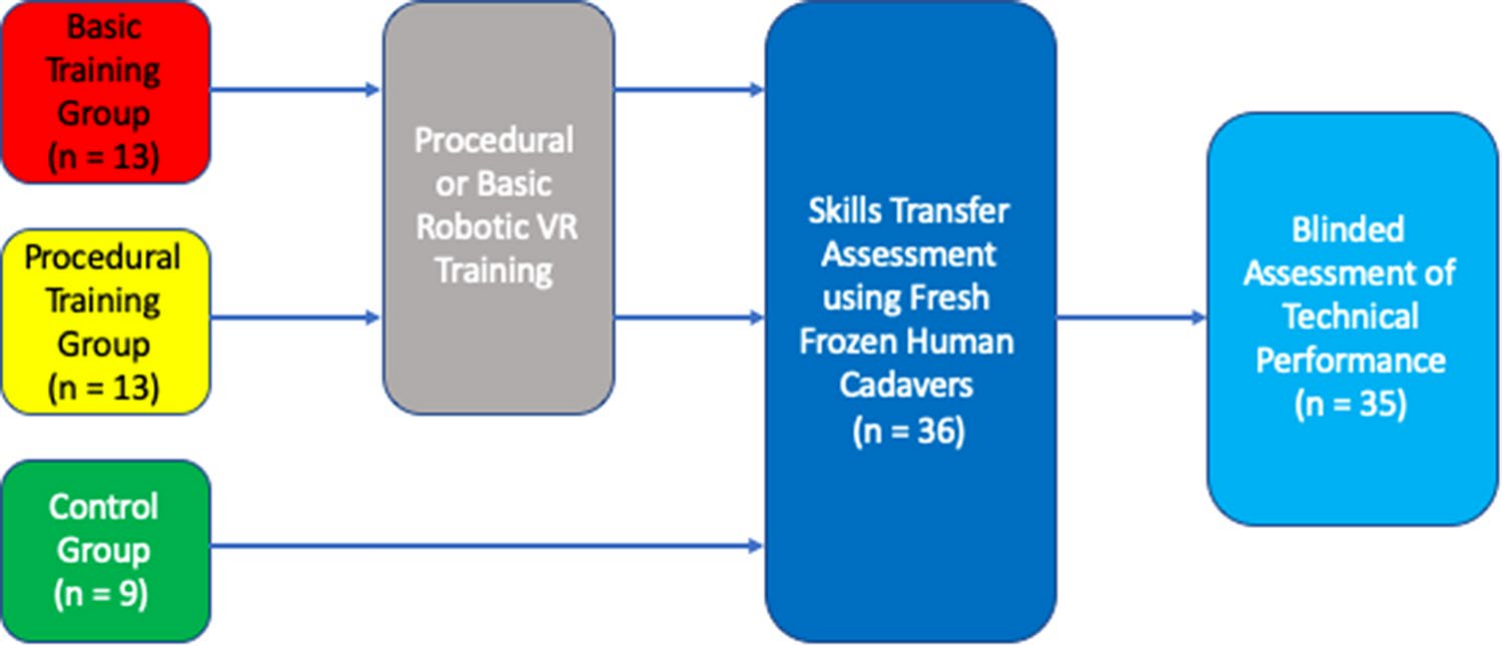
Evaluation of procedural virtual reality simulation training for robotic surgery trial protocol

Initially, all novice participants underwent generic robotic skills training (Fig. 2). This involved completing three Fundamentals of Robotic Surgery (FRS) tasks during a 1-h training session (Ring Tower Transfer, Railroad Track, Vessel Energy Dissection). These tasks were selected to provide the exposure to core robotic skills including endowrist manipulation, camera navigation, dissection and diathermy use. No data were collected during this familiarisation training.

Following familiarisation, all novice participants were randomised using a block randomisation protocol (http://www.randomization.com). Participants were randomised into two groups; procedural VR training or basic VR training (for CONSORT flow diagram see Supplementary Figure 1). For either cohort a programme of structured training was developed based on the simulator training modules. Training followed a competency-based approach whereby participants were encouraged to complete tasks in sequential manner.

The procedural VR cohort underwent training over the course of 5 weeks consisting of 1-h sessions using the guided BND and UVA tasks. During these sessions, ad hoc training and guidance was provided by TA, a study author and expert robotic surgeon. Similarly, proctoring was also provided to basic VR cohort.

The basic VR training group underwent a parallel training programme using the FRS curriculum and a continuous suturing module. Successful completion of each module was determined by competency scores provided by the RobotiX Mentor software.

A further group of novice participants were recruited by open invitation as described above and acted as controls. They did not undergo any robotic skills training prior to the cadaveric performance assessment task.

### Learning curve analysis

Training effects were compared directly through learning curve analysis. Two tasks were selected from each training curriculum. For basic VR training, the Rail Road suturing and Ring Tower Transfer tasks were selected which were compared to BND and UVA (procedural VR). Tasks were carefully selected by authors on the basis of skill that each assessed to ensure they were comparable. Analysis was limited to common metrics to all four modules. Learning curve assessment was limited to the first five attempts. To allow comparison between the different exercises, *Z* scores were calculated.

### Cadaveric performance assessment

Following training, both training groups and the control group underwent an assessment of skills transfer using human fresh frozen cadavers. Cadavers were set up within an “Igloo” disseminated operating room to provide a realistic surgical environment [6]. To complement the procedural training, RARP was used as the assessment task and the cadaver was placed in the Trendelenburg position. The aim was to evaluate the transfer of generic robotic skills, developed through the skills training programme, to the OR. All participants in either the intervention or control cohorts were allocated a 15-min assessment slot. A Da Vinci Si robotic system (Intuitive Surgical, CA) was used. Participants were given a short introduction on using the robot. For each assessment, the participants were guided through steps of a RARP by two study investigators, NR and TA. Complex steps such as the urethrovesical anastomosis were avoided to enable fair analysis of all participants. All participant performances were recorded using the robotic laparoscopic camera. Video recordings for one participant in the basic training cohort were corrupted and their results were therefore excluded from further analysis. Technical performance was evaluated post hoc using the GEARS assessment tool with each performance scored out of a maximum of 30. The assessor was blinded to the participants’ identity or allocation status.

### Statistical analysis

Performance metric data were retrieved from the simulator and *Z* scores calculated. Learning curves were plotted and analysed visually using GraphPad (Prism version 8.4.1, GraphPad Software, La Jolla California USA). Parametric analyses of the cadaveric assessment performance data were undertaken. Statistical analyses were performed using SPSS (IBM SPSS Statistics for Macintosh, Version 25.0, Armonk, NY: IBM Corp).

## Results

26 novice participants were recruited to the study. None had experience in robotic surgery (either live or simulated). Following informed consent all participants underwent baseline training and were then randomised (Fig. 2).

Over the course of five weeks participants underwent training according to their randomisation status. In total, participants completed a 3.0 ± 0.9 h of simulation training (procedural VR group: 2.7 ± 1.2 h; basic VR group: 3.2 ± 0.4 h; *p* = 0.1).

**Fig. 3:**
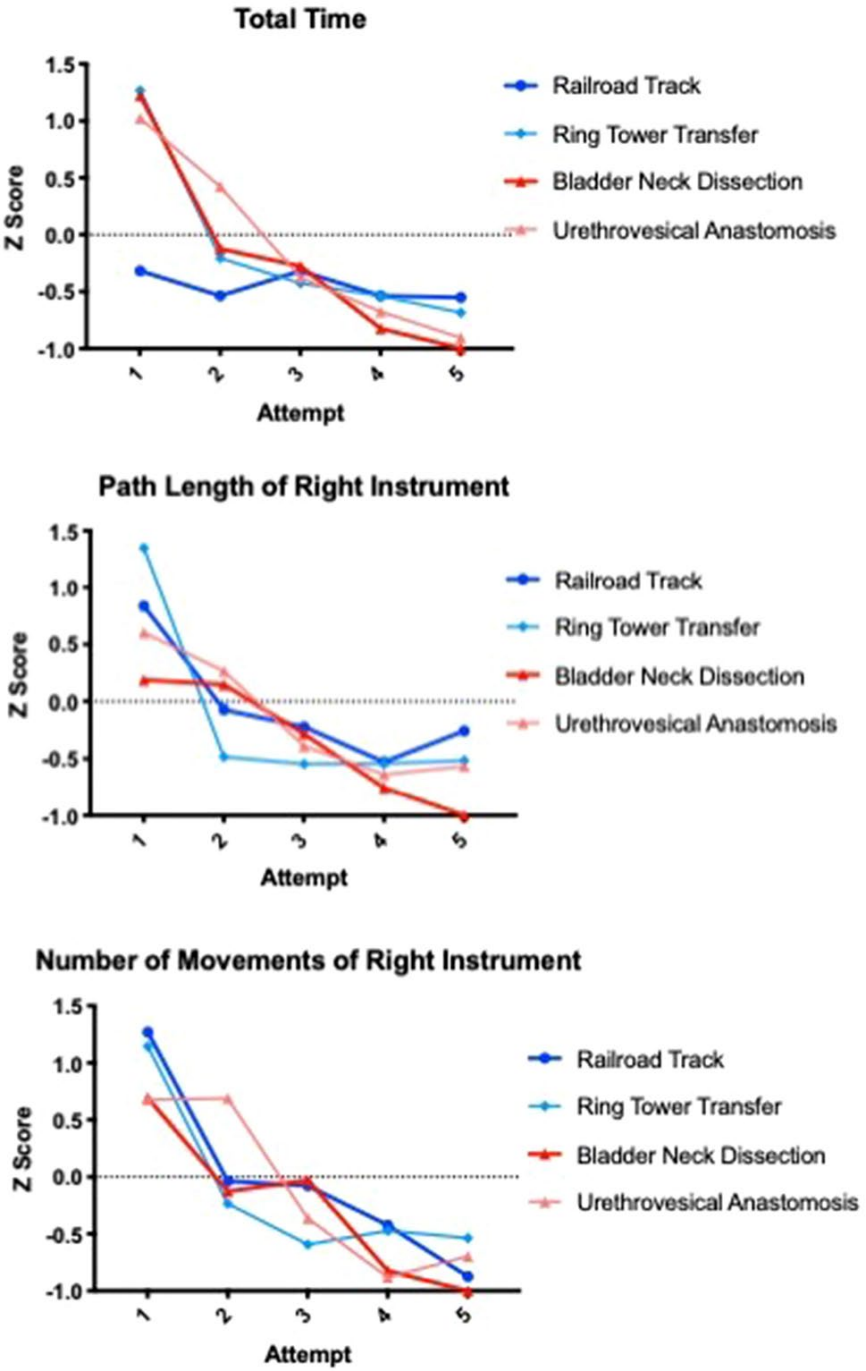
Learning curves for basic and procedural training for total time, number of movements for right instrument and path length for right instrument

Metric data from the simulation training were analysed. Learning curves demonstrated differences between basic and procedural training groups. Overall, there was a noticeable improvement in scores with both simulation techniques particularly during the first three attempts. Training effects were seen most markedly in clutch usage, instrument collisions and the number of movements of left and right instruments. Basic and procedural training both demonstrated improvements particularly in these metrics. Greatest improvements were seen in the procedural (BND and UVA) tasks especially for total time, number of movements for right instrument and path length for right instrument (Fig. 3, see Supplementary Figure 2 for full results).

### Skills transfer assessment on fresh frozen cadavers

**Fig. 4:**
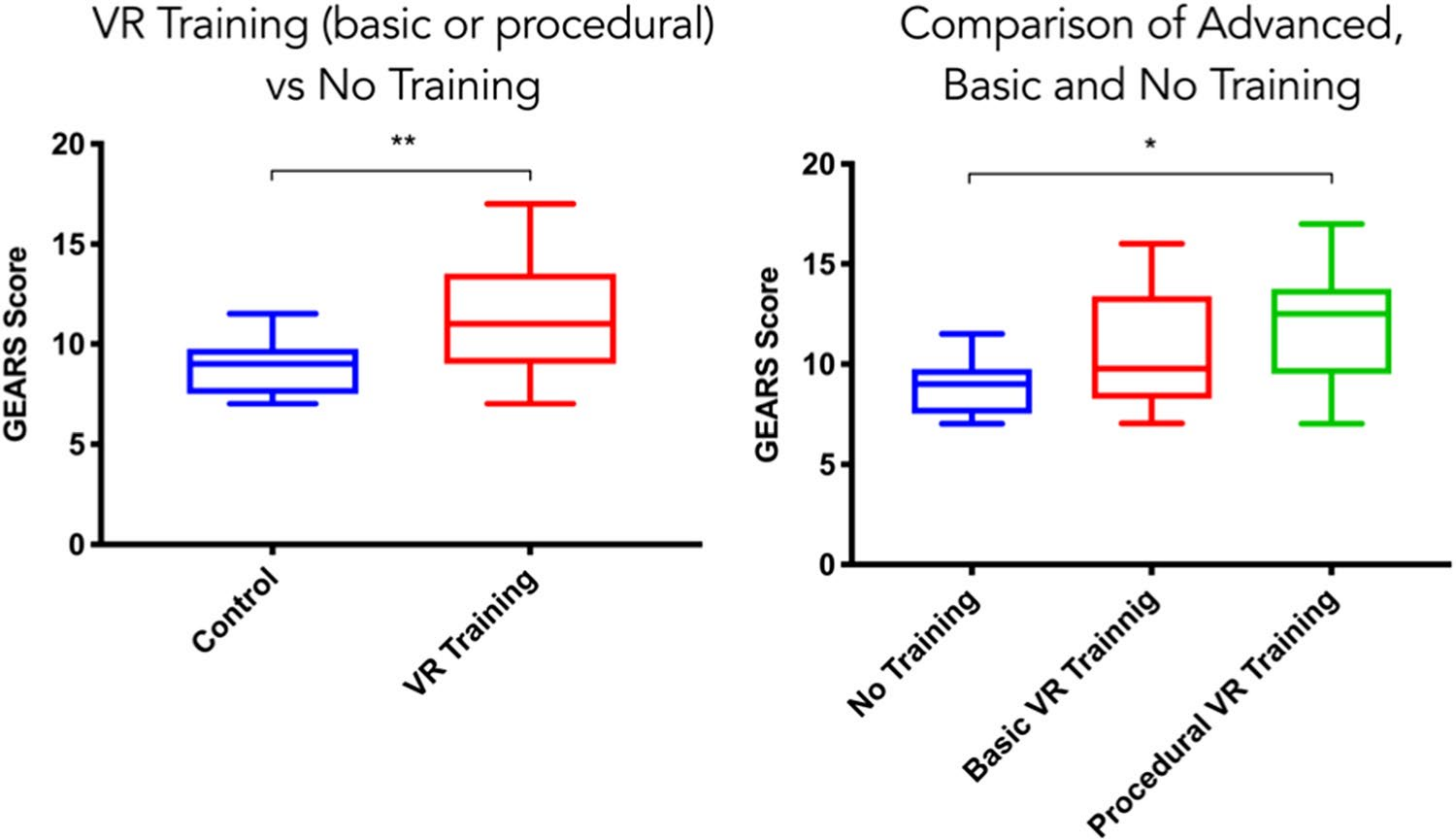
Comparisons of any VR training vs. no training and basic VR, procedural VR and no training

Performance data from 25 study participants who completed the skill transfer assessment were compared to 9 control participants. Any VR training (procedural or basic training) resulted in a significantly higher GEARS score than no training (mean GEARS score 11.3 ± 0.58 vs. 8.8 ± 2.9 *p* = 0.002) (Fig. 4). Procedural training was found to be more effective than either basic or no training; mean GEARS 11.9 ± 2.9 vs. 10.7 ± 2.8 vs. 8.8 ± 1.4, respectively, *p* = 0.03 (Fig. 4).

## Discussion

This randomised controlled trial firstly provides objective evidence that structured VR training (either basic or procedural) is effective in improving robotic surgical skills compared to no training. Importantly, procedural VR training resulted in better skill acquisition and training outcomes than basic VR simulation or no training.

The effectiveness of simulation training is increasingly well established across numerous surgical disciplines. VR simulation for laparoscopic surgery is supported by landmark studies showing that training can translate into improved operative performances [7, 8]. Particularly for a training tool, it may be argued that the consequences or extrapolation inferences of training are one of the most important facets of validity evidence [9]. Direct effects on performance within the OR, either live or simulated, are key for supporting the implementation of simulation tools. Yet, these results do assume further downstream consequences on healthcare outcomes such as patient-reported outcomes. Few studies offer evidence for these outcomes [10].

For robotic surgery, basic VR simulators have undergone extensive validation but important evidence for the consequences of training remains limited [11]. Culligan et al. [12] demonstrated that completing a training programme using the dVSS simulator led to successful completion of a supravesical hysterectomy equivalent to experienced robotic surgeons. Hung et al. [13] also assessed skills transfer effect of basic skills VR training using an ex vivo animal model but found no significance difference compared to a non-training group. The evidence from our study adds to the body of evidence supporting the role of basic VR simulation.

In comparison, evidence for procedural VR simulators in robotic surgery is very limited. Of the six VR simulators commercially available, three offer procedural-based training (dV-Trainer, Mimic Technologies, USA; RobotiX Mentor, 3D Systems, USA, USA; RoSS, Simulated Surgical Systems, USA; SEP Robot, SimSurgery, Norway). Initial validation of the RobotiX Mentor has been reported supporting its content [14]. This current study builds on this initial experience. Using a randomised design, our data show beneficial effects of training both on performance on the VR simulator as assessed through learning curves and the transfer of learning effects have been shown. The cadaveric task was chosen to both provide an accurate assessment of operative robotic skill in line with the training from the RobotiX Mentor. Importantly, greater benefit was seen with procedural-based training. Whilst this is the first direct comparison of procedural and basic VR training, data from prior studies do suggest greater benefit with procedural-based training [15]. The reasons for this are not clear but the results of this study have important implications for surgical training. Anecdotal feedback from participants was that the procedural training was more enjoyable than the abstract basic skills tasks. Such more clinically relevant training may help to motivate participants and focus their training.

Limitations to this study need to be considered. The results need to be reviewed in the context of the trial design and the limited numbers of participants. Closer correlations between educational interventions and outcomes are found with more experienced participants as well as with specific clinical encounters rather than general impressions [10]. This study may therefore underestimate the beneficial effects of procedural VR. Further analysis of the outcomes of training in a larger cohort of trainees is also required to be able to assess the value of procedural over basic VR training. It is also important to recognise that validity evidence is specific to the context in which assessments were undertaken [16]. Outcomes from this study result from a period of structured training, however, the assessment should not be considered summative. Assessment time was set to 15 min to balance adequate assessment with resource limitations. It has been shown that 5-min recordings are adequate for GEARS assessment by both expert and amateur raters [17]. Further research is required to evaluate the role of procedural VR for high stakes assessments. Likewise, this study used RARP performed on human cadavers in a simulated operating room environment as a surrogate for live surgery. Disseminated surgical environments provide a realistic the reliability of procedural training needs to be established through additional studies. Development of objective competency criteria will also be required to support the integration of procedural VR into robotic curricula.

This study aimed to evaluate the role of procedural VR training for robotic surgery. Procedural VR training has been found to be more effective than both no training and basic VR simulation. These results offer important considerations for the development of robotic surgical training programmes. In addition, such training offers the possibility of developing more advanced surgical competencies than the basic motor skills that are the focus of current VR programmes. Including non-technical skill such as decision making and judgement will become an increasingly important aspect of surgical curriculum development.

## Electronic supplementary material

Below is the link to the electronic supplementary material.

Supplementary file1 (DOCX 60 KB)

Supplementary file2 (JPG 2932 KB)
