# Supplementary material for: Procedural virtual reality simulation training for robotic surgery: a randomised controlled trial
Source: Surg Endosc. 2021 Jan 4;35(12):6897–902. doi: 10.1007/s00464-020-08197-w (PMC8599326; doi:10.1007/s00464-020-08197-w)
Supplement: Supplementary file 1 — Supplementary file1 (DOCX 60 KB) [file 464_2020_8197_MOESM1_ESM.docx]

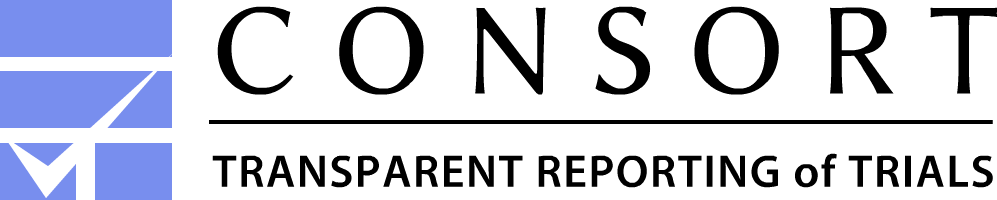


**CONSORT 2010 Flow Diagram**

Control Participants (n=9)

Control (n=9)

♦ Excluded from analysis (n=0)

## Enrollment

Analysed (n=13)
♦ Excluded from analysis (n=0)

Lost to follow-up (give reasons) (n= 0)

Discontinued intervention (n=0)

Allocated to procedural VR training (n=13)

♦ Received allocated intervention (n=13)

♦ Did not receive allocated intervention (n=0)

Analysed (n=12)
♦ Excluded from analysis (corrupted video files) (n=1)

Lost to follow-up (give reasons) (n=0)

Discontinued intervention (n=0)

Allocated to basic VR training (n= 13)

♦ Received allocated intervention (n=13)

♦ Did not receive allocated intervention (n=0)

## Follow-Up

## Analysis

## Allocation

Randomized (n=26)

Assessed for eligibility (n= 26)
