# Supplementary figures and images for: Procedural virtual reality simulation training for robotic surgery: a randomised controlled trial
Source: Surg Endosc. 2021 Jan 4;35(12):6897–902. doi: 10.1007/s00464-020-08197-w (PMC8599326; doi:10.1007/s00464-020-08197-w)

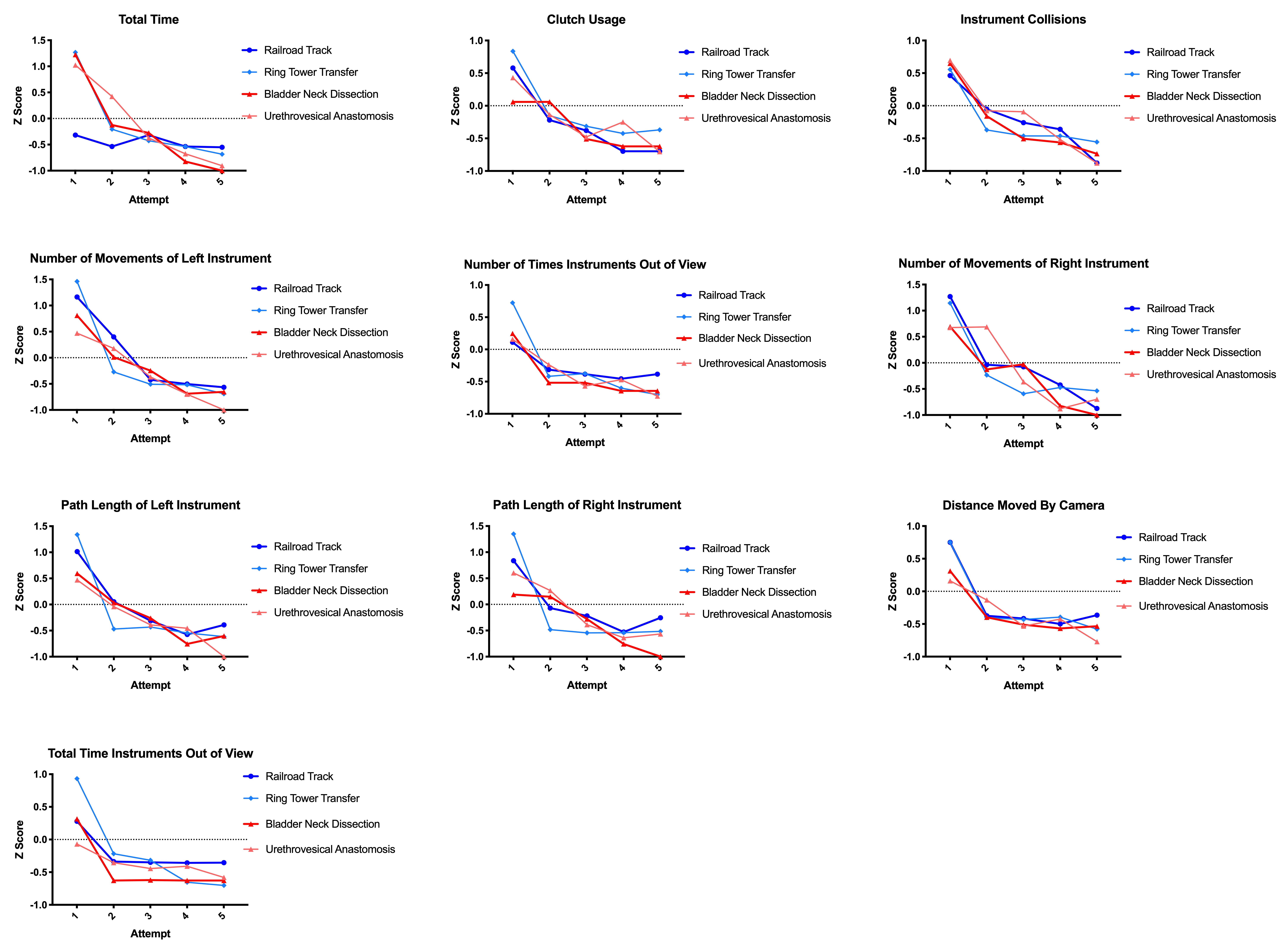

Supplement: Supplementary file 2 — Supplementary file2 (JPG 2932 KB) [file 464_2020_8197_MOESM2_ESM.jpg]
